# Bladder glandular cystitis causing renal dysfunction: A case report

**DOI:** 10.1016/j.ijscr.2025.111171

**Published:** 2025-03-20

**Authors:** Salim Lachkar, Ahmed Ibrahimi, Imad Boualaoui, Hachem El Sayegh, Yassine Nouini

**Affiliations:** Department of Urology A, Ibn Sina University Hospital, Rabat, Morocco

**Keywords:** Glandular cystitis, Urinary tract disorders, Intestinal metaplasia, Urothelial metaplasia, Case report

## Abstract

**Introduction:**

Glandular cystitis is a rare bladder condition with urothelial metaplasia, often linked to chronic irritation and mimicking malignant tumors. Diagnosis is histopathological, utilizing CK7 and CK20 markers. While usually benign, some cases may be associated with carcinoma, requiring long-term surveillance.

**Presentation of case:**

Mrs. M, a 68-year-old active smoker, presented with acute oliguria, bilateral flank pain, and other urinary symptoms. Lab results indicated severe acute kidney injury, and a CT scan revealed bilateral hydronephrosis and a 4 cm bladder lesion. After stabilization with hemodialysis, nephrostomies were placed. Two weeks later, transurethral resection showed an atypical bladder lesion, and histopathology confirmed intestinal glandular cystitis. Surveillance cystoscopies were scheduled, with no recurrence at three years. Mrs. M showed significant symptom relief and improved quality of life, as assessed by the FLZM questionnaire.

**Discussion:**

GC is a rare bladder condition with two forms: glandular (most common) and intestinal metaplasia, which may mimic malignant tumors. Diagnosis relies on cystoscopy, histopathology, and immunohistochemical markers (CK7 and CK20). Non-urological exams, like colonoscopy, rule out systemic involvement. Most cases are benign, but some may be linked to carcinoma in situ or adenocarcinoma. Treatment includes conservative therapies, pharmacologic agents, and transurethral resection for pseudotumoral forms. Fucoidan shows promise as a therapeutic agent. Invasive surgeries like cystectomy or ureteral reimplantation are considered for recurrent cases. Surveillance is necessary due to malignant transformation potential.

**Conclusion:**

GC, though rare, demands early recognition, accurate diagnosis, and tailored management to prevent complications. This case emphasizes the importance of vigilant follow-up and care.

## Highlights

• Glandular cystitis: Rare bladder condition with urothelial metaplasia.
• Diagnosis confirmed through histopathology and immunohistochemistry (CK7, CK20).
• Conservative and surgical treatments for pseudotumoral forms of GC.
• Surveillance needed due to potential malignant transformation.

## 1 Introduction

Glandular cystitis (GC) is a rare condition characterized by vesical urothelium metaplasia at Von Brunn's nests, predominantly affecting males and linked to chronic bladder irritation [1]. It can mimic malignant bladder tumors, presenting with nonspecific symptoms [2]. Diagnosis is made via transurethral resection (TURB), with histopathology showing glandular or intestinal forms. Immunohistochemical markers aid in diagnosis [3]. While most cases are benign, some may be associated with carcinoma in situ or invasive adenocarcinoma, necessitating long-term surveillance and multidisciplinary care [4]. This case emphasizes the importance of early recognition and management. To our knowledge, this is the second published case of renal insufficiency secondary to ureteral obstruction caused by vesical glandular cystitis [5].

## 2 Case presentation

Mrs. M, a 68-year-old active smoker with no significant medical history, presented with acute oliguria (<300 mL urine) without the sensation of needing to urinate and bilateral flank pain, more severe on the left, suggestive of renal colic. She also reported abdominal bloating, nausea, generalized weakness, dysuria, nocturnal frequency, urinary urgency, pelvic pain, and intermittent hematuria.

On examination, she had respiratory distress, dyspnea, tachycardia (HR 110 bpm), and elevated blood pressure (160/95 mmHg), likely due to volume overload, as indicated by soft, pitting edema in the lower extremities. Abdominal exam showed no palpable bladder, bilateral flank tenderness, and lung auscultation revealed fine crackles, indicating pulmonary congestion. There was no fever or weight loss.

Laboratory tests showed severe acute kidney injury with creatinine of 12.4 mg/dL (normal: 0.6–1.2 mg/dL), urea of 380 mg/dL (normal: 15–40 mg/dL), hyperkalemia at 7.3 mmol/L (normal: 3.5–5.0 mmol/L), and metabolic acidosis with bicarbonates at 6 mmol/L (normal: 22–28 mmol/L). Blood gas analysis revealed a pH of 7.19 (normal: 7.35–7.45), indicating life-threatening metabolic derangements.

**Fig. 1:**
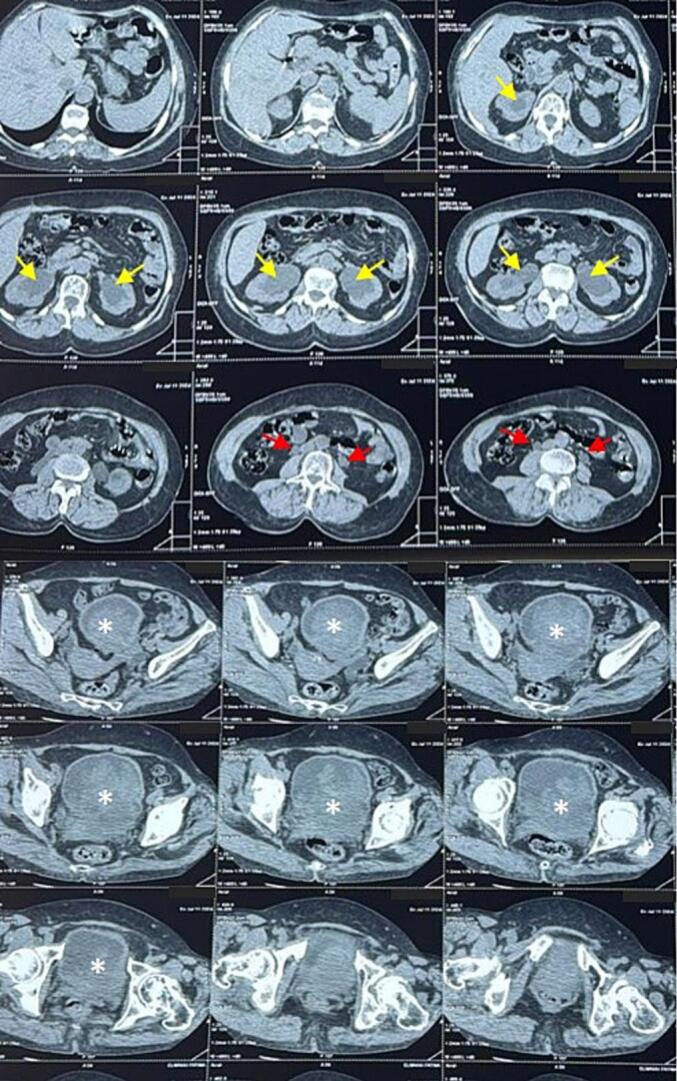
Non-contrast CT scan: Bilateral ureterohydronephrosis with symmetrical pyelocaliceal (yellow arrow) and ureteral dilation (red arrow) upstream of a 4 cm trigonal bladder lesion (white asterisk). (For interpretation of the references to colour in this figure legend, the reader is referred to the web version of this article.)

A non-contrast abdominal and pelvic CT scan revealed severe bilateral upper urinary tract hydronephrosis with marked dilation of the renal collecting systems, particularly on the left. The kidneys were well-preserved in size and differentiation, indicating acute obstruction. A 4 cm lesion in the trigonal region of the bladder, highly suspicious for a tumor, was identified as the cause of bladder outlet obstruction. Fig. 1.

**Fig. 2:**
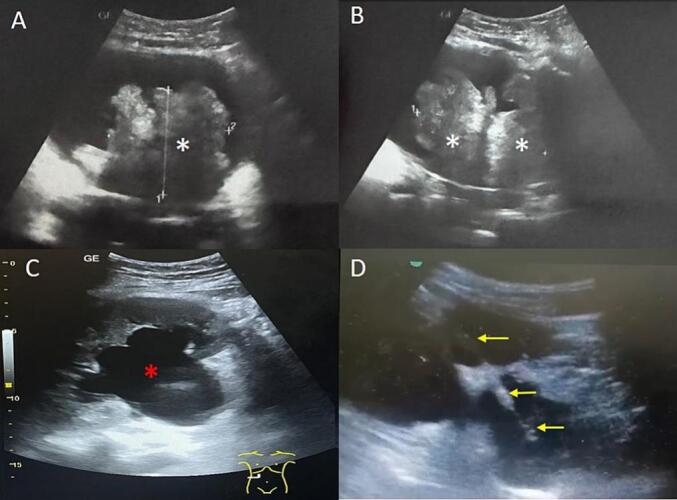
Renovesical ultrasound guiding nephrostomy placement: (A, B) Bladder lesion (white asterisk); (C) Pyelocaliceal dilation (red asterisk); (D) Puncture needle trajectory (yellow arrow). (For interpretation of the references to colour in this figure legend, the reader is referred to the web version of this article.)

Following stabilization with emergency hemodialysis, bilateral nephrostomies were placed under ultrasound guidance to minimize the risk of neoplastic cell seeding, compared to double J stents. Fig. 2 One week later, both clinical and laboratory values had normalized.

**Fig. 3:**
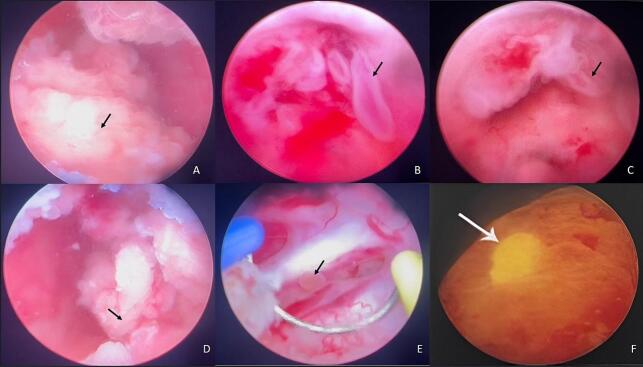
Endoscopic examination: (A) Lesion involving the right ureteral orifice (black arrow); (B, C) Voluminous, atypical, and irregular lesion with a bullous aspect (black arrow); (D) Extension to the left ureteral orifice (black arrow); (E) Inflamed and hyperemic mucosa with bullous lesions (black arrow); (F) Closer view using Clara and Chroma settings for enhanced contrast.

Two weeks later, transurethral resection of the bladder lesion was performed. Endoscopic examination revealed an atypical lesion with an irregular, slightly raised appearance, showing mucosal inflammation and hyperplasia rather than the typical papillary configuration of urothelial carcinoma. Fig. 3 The ureteral orifices, which were encroached upon, were released through resection, allowing subsequent removal of the nephrostomies.

**Fig. 4:**
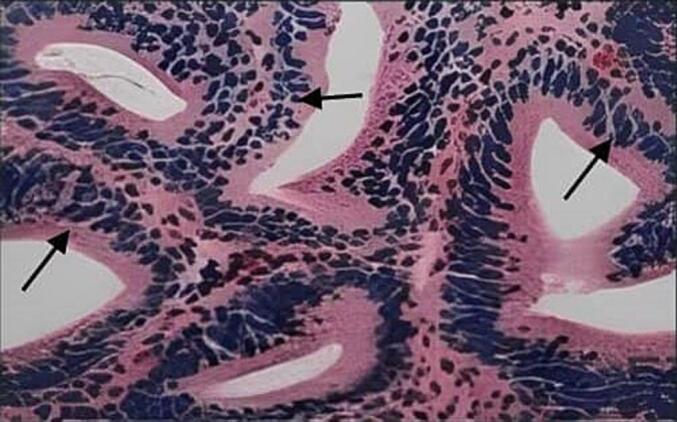
Histological appearance: Cylindrical cells lining the glandular lumen surrounded by layers of urothelial cells (black arrow).

Histopathological examination identified intestinal glandular cystitis, a rare bladder inflammation characterized by goblet cells and intestinal-type glands in the bladder wall. Immunohistochemical staining revealed strong positivity for CK7 and CK20, markers commonly associated with glandular cystitis. Fig. 4.

Colonoscopy and gastrointestinal imaging ruled out malignancies, confirming that the intestinal metaplasia was confined to the bladder.

In the absence of consensus guidelines for the follow-up of pseudo-tumoral forms of glandular cystitis, we opted for surveillance cystoscopies every six months for the first two years, followed by annual cystoscopies for three years. At three years, the bladder remained healthy with no recurrence of lesions.

Following surgery, Mrs. M reported significant symptom relief and a 40 % improvement in quality of life, as assessed by the FLZM (*Fragen zur Lebenszufriedenheit*) questionnaire, routinely used in our department [6]. Her score increased from 40/100 to 80/100, with specific scores of 17/20 for general health, 16/20 for physical capacity, 15/20 for emotional state, 17/20 for satisfaction with social relations, and 15/20 for overall life satisfaction. In terms of symptoms, bilateral flank pain, abdominal bloating, nausea, and intermittent hematuria resolved completely, while dysuria, nocturnal frequency, and urinary urgency showed substantial improvement. Unfortunately, pelvic pain persisted.

## 3 Discussion

GC is an uncommon metaplastic transformation of the bladder epithelium at Von Brunn's nests. Its etiology is multifactorial and poorly understood, often linked to chronic bladder irritation from recurrent infections, bladder stones, or prolonged catheterization. Other contributing factors, such as avitaminosis, medications, and hormonal imbalances, have been proposed [1].

The exact incidence of GC remains unclear due to its rarity and often asymptomatic nature. In a study of 100 histopathological cases from transurethral resection of the bladder for various causes, 3 % were diagnosed with GC [3]. It predominantly affects middle-aged men, though cases in women and children have also been reported [7].

To our knowledge, this is the second reported case of renal insufficiency due to ureteral obstruction caused by vesical glandular cystitis, the first being a Spanish case published on PubMed in 2003 [5]. Furthermore, fewer than 30 cases of glandular cystitis with ureterohydronephrosis, without renal insufficiency, have been reported [[7], [8], [9]].

Histologically, GC primarily presents in two forms [2]. The glandular form, the most common, is characterized by mucin-secreting changes in the bladder epithelium and is often asymptomatic. The less frequent intestinal metaplasia form features glandular structures lined by colonic mucinous cells proliferating within the superficial lamina propria. Rarely, GC may exhibit gastric or prostatic metaplasia [1]. Immunohistochemical staining is crucial for diagnosing GC and differentiating it from other bladder lesions, especially adenocarcinoma. GC typically shows CK7 positivity and CK20 negativity, whereas adenocarcinoma expresses both markers [3].

GC's clinical presentation varies widely. Many cases are asymptomatic, but when symptoms occur, they mainly include lower urinary tract irritative symptoms, macroscopic hematuria, and, occasionally, lower back pain due to meatal involvement [10]. GC is a significant cause of bladder pain syndrome [2]. Physical examinations are usually unremarkable, although pelvic shielding can sometimes be detected in cases of associated pelvicalyceal lipomatosis [8].

Imaging studies, such as ultrasound and uro-CT, are non-specific but may mimic bladder carcinoma, showing trigonal masses or bladder wall thickening. MRI does not offer better diagnostic yield and is reserved for cases where CT is contraindicated [11]. The UHN found in our case is a radiological sign described in GC [9]. In a study of 767 UHN cases, excluding ureteral calculi or upper urinary tract tumors, UHN associated with pelvic lipomatosis and/or cystitis glandularis represented 5 % of cases [12]. In cases of associated pelvicalyceal lipomatosis, MRI with “FAT SAT” sequences plays a pivotal role in revealing excess pelvic fat [12].

Cystoscopy often reveals pseudotumoral, edematous cysts ranging from 1 to 15 mm in diameter. Blue light cystoscopy improves detection [3]. However, histopathological examination remains the definitive diagnostic tool, identifying cylindrical glandular tissue.

Non-urological exams, such as colonoscopy and gastrointestinal imaging, are crucial for diagnosing GC with suspected intestinal metaplasia [13]. Colonoscopy rules out gastrointestinal malignancies, like colorectal cancer, which can present similarly. It also confirms if intestinal metaplasia is confined to the bladder, excluding systemic involvement [4]. CT and MRI scans help differentiate GC from conditions like inflammatory bowel disease or colorectal cancer by detecting signs such as enlarged lymph nodes or bowel wall thickening, with MRI providing detailed visualization of pelvic and abdominal structures [13]. Even if not specific to GC, these exams ensure a more accurate urological evaluation by excluding malignancy and gastrointestinal issues.

Differentiation from other bladder conditions is essential. Chronic cystitis shares similar symptoms but lacks GC's glandular growth [1]. Bladder carcinoma, which can cause plaques or masses, requires histological confirmation to rule out malignancy [4]. Leukoplakia of the bladder can resemble GC cystoscopically but features stratified squamous epithelium with keratinization [14]. In endemic regions, schistosomiasis causes inflammatory plaques distinct from GC due to the presence of parasitic eggs and other findings [2]. Accurate diagnosis relies on cystoscopic and histopathological evaluation.

GC management depends on disease extent and symptoms. Because of renal insufficiency, we performed bilateral nephrostomies, as in another case of glandular cystitis [5]. Treatment is multimodal, focusing on addressing bladder irritation sources [15]. Conservative options include dietary adjustments, behavioral therapy and physical therapy. Pharmacologic treatments like amitriptyline or pentosan polysulfate sodium may help [15]. Endovesical instillations using agents such as anti-angiogenics, hydrocortisone, or dimethyl sulfoxide have been reported [10]. In contrast, techniques like bladder hydrodistension or botulinum toxin A injections, recommended for BPS/IC, are not commonly used for GC [16].

The potential of fucoidan as a therapeutic agent for GC represents a new promising avenue, targeting six core mechanisms involved in its anti-CG effects [17]. Fucoidan acts primarily through modulating biological processes related to inflammatory stress, immune responses, and immune infiltration. Key signaling pathways include IL-17, Th17 cell differentiation, T-cell receptor signaling, TNF, cytokine-cytokine receptor interactions, Toll-like receptors, and NF-kappa B signaling [17]. However, further experimental and clinical validation is required to confirm these findings.

For pseudotumoral forms, transurethral resection (TURB) is typically sufficient, providing complete tumor removal and symptom relief while reducing recurrence risk [15]. Laser YAG photocoagulation has also been reported [8]. We opted for TURB in our case with significant improvement, as in another case of glandular cystitis with renal insufficiency [5].

More invasive surgeries, such as bilateral ureteral reimplantation, enterocystoplasty, or cystectomy, are reserved for extensive forms, recurrent cases, or those involving upper urinary tract dilation [18]. A systematic review on surgery for conservative-treatment-resistant BPS/IC reported symptom relief in 77.2 % of cases, though with a complication rate of 26.5 % [16]. These findings highlight the need for larger studies to refine patient selection and optimize surgical strategies. Radical cystectomy using fertility-preserving techniques can maintain fertility and quality of life. One notable case describes a successful pregnancy following robot-assisted fertility-preserving cystectomy with an ileal orthotopic neobladder in a 43-year-old woman [19].

GC's natural course remains debated [2]. While it is usually a benign lesion, the florid form may have precancerous potential, progressing to adenocarcinoma in rare cases with persistent chronic irritation [4]. Pseudotumoral forms require regular surveillance through imaging and cystoscopy [1].

This case report of a rare presentation of GC contributes to the limited literature on the condition. However, its limitations include reliance on a single case, the lack of long-term follow-up, and the absence of consensus guidelines for managing this rare disorder.

## 4 Conclusion

CG, a rare condition that can cause BPS/IC, lacks consensus guidelines for management. Diagnosis relies on immunohistochemical staining (CK7, CK20). Treatment depends on disease extent, and vigilant surveillance is necessary, especially in cases with intestinal metaplasia, to monitor for potential malignancy.

## Abbreviations

TURB: Transurethral resection of the bladder
BPS/IC: Bladder pain syndrome/interstitial cystitis
GC: Glandular cystitis

## Consent

The patient provided informed consent after receiving detailed information regarding the study and its implications.

## Ethical approval

Ethical approval was obtained from our institution. Informed consent was provided by the patient for the publication of case details and images.

## Methods

This case report has been conducted and reported in accordance with the SCARE (Surgical CAse REport) guidelines [20].

## Funding resources

This research and the publication expenses were solely supported by the author, and no specific grant was received from any public, commercial, or non-profit sectors.

## Declaration of competing interest

None.

## Data Availability

Data associated with the study has not been deposited into a publicly available repository. The data that has been used is confidential.
